# Picroside II Isolated from *Pseudolysimachion rotundum var. subintegrum* Inhibits Glucocorticoid Refractory Serum Amyloid A (SAA) Expression and SAA-induced IL-33 Secretion

**DOI:** 10.3390/molecules24102020

**Published:** 2019-05-27

**Authors:** Kiram Lee, Jin Choi, Bo Kyong Choi, Young-Mi Gu, Hyung Won Ryu, Sei-Ryang Oh, Hyun-Jun Lee

**Affiliations:** 1 Natural Medicine Research Center, Korea Research Institute of Bioscience and Biotechnology (KRIBB), 30 Yeongudanji-ro, Ochang-eup, Cheongwon-gu, Cheongju, Chungbuk 28116, Korea; krl05@kribb.re.kr (K.L.); chojinsg@gail.com (J.C.); bkchoi@progen.co.kr (B.K.C.); kuy823@kribb.re.kr (Y.-M.G.); ryuhw@kribb.re.kr (H.W.R.); seiryang@kribb.re.kr (S.-R.O.); 2 Department of Biomolecular Science, University of Science & Technology (UST), Daejeon 341113, Korea

**Keywords:** Picroside II, *Pseudolysimachion rotundum* var. subintegrum, serum amyloid A, IL-33, steroid-resistance, airway epithelial cells

## Abstract

Chronic obstructive pulmonary disease (COPD) is a major inflammatory lung disease characterized by irreversible and progressive airflow obstruction. Although corticosteroids are often used to reduce inflammation, steroid therapies are insufficient in patients with refractory COPD. Both serum amyloid A (SAA) and IL-33 have been implicated in the pathology of steroid-resistant lung inflammation. Picroside II isolated from *Pseudolysimachion rotundum* var. *subintegrum* *(Plantaginaceae)* is a major bioactive component of YPL-001, which has completed phase-2a clinical trials in chronic obstructive pulmonary disease patients. In this study, we investigated whether picroside II is effective in treating steroid refractory lung inflammation via the inhibition of the SAA-IL-33 axis. Picroside II inhibited LPS-induced *SAA1* expression in human monocytes, which are resistant to steroids. SAA induced the secretion of IL-33 without involving cell necrosis. Picroside II, but not dexamethasone effectively inhibited SAA-induced IL-33 expression and secretion. The inhibitory effect by picroside II was mediated by suppressing the mitogen-activated protein kinase (MAPK) p38, ERK1/2, and nuclear factor-κB pathways. Our results suggest that picroside II negatively modulates the SAA-IL-33 axis that has been implicated in steroid-resistant lung inflammation. These findings provide valuable information for the development of picroside II as an alternative therapeutic agent against steroid refractory lung inflammation in COPD.

## 1. Introduction

Chronic obstructive pulmonary disease (COPD) is a pulmonary disease characterized by irreversible and progressive airflow obstruction [1]. COPD is a major chronic inflammatory lung disease with increasing prevalence [2]. At present, COPD is the fourth leading cause of death worldwide [3], and no drug treatments are currently available to cure or considerably decrease COPD mortality [4].

Currently, steroids are the most common and effective anti-inflammatory drugs. However, refractory COPD patients respond poorly to steroid treatment, exhibiting persistent airway inflammation [5]. The resistance to the anti-inflammatory effects of glucocorticoids in COPD is a continuing clinical problem. Therefore, there is a need to identify novel drugs to prevent steroid-resistant lung inflammation. A recent article reported that the increase in the serum level of serum amyloid A (SAA) is associated with glucocorticoid resistance [6].

Inducible SAA (encoded by *SAA1* and *SAA2* genes) is one of the major acute-phase proteins released to circulation in response to inflammation, infection, and injury [7]. Many clinical studies have shown that SAA levels in serum predict the severity of acute exacerbations of COPD [8,9,10]. These findings suggest that SAA is an important inflammatory mediator in the lungs. SAA has been known to stimulate the release of cytokines including IL-23, IL-1β, TNF-α, and IL-8 in specific cell types [11,12,13]. However, the relationship between SAA and IL-33 secretion in airway epithelial cells has not been explored.

Interleukin-33 (IL-33), a recent addition to the IL-1 family, is a critical mediator of type-2 immune responses. Elevated IL-33 concentrations in the nuclei of airway epithelial cells are observed in patients with COPD [14]. Moreover, IL-33 has been implicated as a steroid-resistant mediator that promotes airway remodeling in patients with severe therapy-resistant asthma [15]. Thus, blocking IL-33 would be clinically beneficial for treating lung inflammation.

*Pseudolysimachion rotundum* var. *subintegrum (Plantaginaceae)* has been used as an Asian traditional medicine for the treatment of inflammatory diseases [16]. YPL-001 (drug substance) derived from this herb has successfully completed phase-2a clinical trials in COPD patients (https://clinicaltrialsgov/). Previously, we reported that verproside, a major catalpol-related compound in this plant, showed anti-inflammatory, anti-oxidant, and anti-asthmatic activities [16,17]. Verproside is predominantly metabolized to picroside II in vivo [18]. Recently, we demonstrated that picroside II has therapeutic potential for allergic asthma in HDM-induced asthmatic mice [19]. However, little has been reported about whether picroside II is effective in treating steroid-resistant lung inflammation in COPD.

Here, we demonstrated for the first time that picroside II inhibits LPS-induced *SAA1* expression in human monocytes and SAA-induced IL-33 expression/secretion in human airway epithelial cells via the inhibition of the MAPK and NF-κB pathways. These results suggest that picroside II may have beneficial effects in the treatment of steroid-resistant lung inflammation.

## 2. Results

### 2.1. Picroside II Inhibits LPS-Induced SAA1 Expression in Human Monocytes

**Figure 1:**
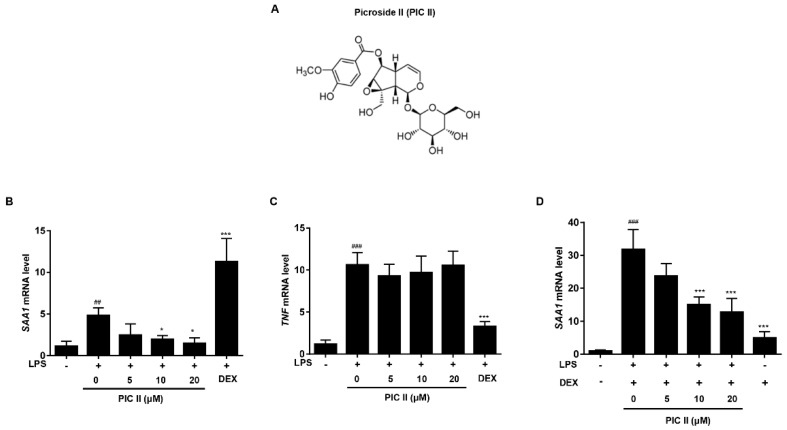
The inhibitory effect of picroside II on LPS-induced SAA expression in THP-1 cells. (**A**) Structure of picroside II (PIC II). (**B**,**C**) THP-1 cells were pretreated with indicated concentrations of PIC II or DEX (1 μM) for 1h and then stimulated with LPS (0.2 μg/mL) for 6 h. The mRNA expressions of *SAA1* (**B**) and *TNF* (**C**) were measured by real-time RT-PCR. (**D**) THP-1 cells were pretreated with indicated concentrations of PIC II for 1h and then stimulated with LPS (0.2 μg/mL) plus DEX (1 μM) for 6 h. The mRNA expression of *SAA1* was measured by real-time RT-PCR. ^##^ *p* < 0.01 and ^###^ *p* < 0.001, compared with medium alone; *p < 0.05 and *** *p* < 0.001, compared with LPS alone.

Extrahepatic SAA is mainly produced by monocytes/macrophages and plays a role in local responses to injury and inflammation [20]. It has been shown that DEX induces SAA expression on its own and synergistically increases LPS-induced SAA expression in THP-1 cells [6]. Thus, we investigated the effect of picroside II on *SAA1* expression using a THP-1 human monocyte cell line. We previously showed that picroside II did not affect cell viability at concentrations up to 20 μM [19]. We first compared the effects of picroside II and DEX on LPS-induced expression of *SAA1* mRNA. Picroside II suppressed *SAA1* mRNA expression induced by LPS in a dose-dependent manner. In contrast, DEX enhanced LPS-induced *SAA1* mRNA (Figure 1B). The reverse was observed with TNFα expression. Picroside II failed to inhibit LPS-induced TNFα expression, while DEX effectively suppressed the gene induction (Figure 1C). We next investigated whether picroside II also inhibits the expression of *SAA1* mRNA induced by LPS plus DEX. As shown in Figure 1D, picroside II reduced the level of *SAA1* mRNA expression induced by LPS plus DEX treatments in a dose-dependent manner. Similar results were obtained when we used β-actin as reference gene (Supplementary Figure S1A,B). Taken together, this data demonstrates that picroside II effectively inhibits LPS-induced *SAA1* mRNA expression that is resistant to DEX in THP-1 cells.

### 2.2. SAA Induces Expression and Secretion of IL-33 in Human Airway Epithelial Cells

**Figure 2:**
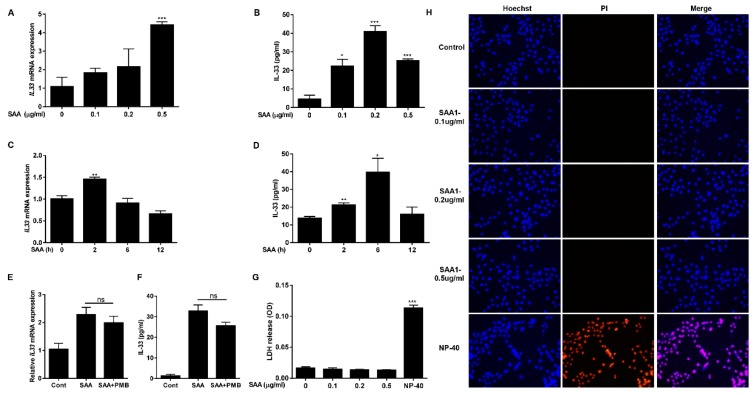
SAA induces the transcription and secretion of IL-33 from NCI-H292 cells in a non-cytotoxic manner. (**A**,**B**) NCI-H292 cells were stimulated with 0.1-0.5 μg/mL of SAA for 6 h. The cells were then analyzed (**A**) for *IL33* mRNA levels by real-time RT-PCR and (**B**) for IL-33 protein production by ELISA. (**C**,**D**) NCI-H292 cells were treated with (**C**) SAA (0.5 μg/mL) to examine *IL33* gene expression by real-time RT-PCR, or treated with (**D**) SAA (0.2 μg/mL) for the indicated periods of time to measure IL-33 protein production by ELISA. (**E**,**F**) Before addition of SAA (0.2 μg/mL, 6 h), NCI-H292 cells were preincubated with PMB (10 μg/mL) for 1h at 37°C, and (**E**) analyzed for *IL33* mRNA levels by real-time RT-PCR, or (**F**) IL-33 protein production by ELISA. (**G**,**H**) NCI-H292 cells were stimulated with indicated SAA concentration or 0.5% NP40 for 6 h. (**G**) Cell-free media were analyzed for LDH activity. (**H**) Propidium iodide (PI)-stained necrotic cells (red) and hoechst 33,342 (Hoechst)-stained living cells (blue) were observed in fluorescence photomicroscope (magnification, ×200).* *p* < 0.05, ** *p* < 0.01, and *** *p* < 0.001, compared with medium alone.

Before addressing the suppressive effect of picroside II on the SAA/IL-33 axis, we first examined whether recombinant human SAA affects the gene expression and secretion of IL-33 in NCI-H292 human airway epithelial cells. As shown in Figure 2A, SAA dose-dependently upregulated *IL33* gene expression. The secretion of IL-33 was detected at a SAA concentration of 0.1 μg/mL and reached a maximal level at a SAA concentration of 0.2 μg/mL (Figure 2B). Next, we examined the time course of IL-33 gene expression and protein secretion induced by SAA. After 2 h treatment with SAA, *IL33* mRNA expression level was increased significantly and then decreased after 6 h (Figure 2C). SAA induced IL-33 release started to increase after 2 h of incubation and increased further until the 6 h time point, returning to normal levels after 12 h (Figure 2D). These results showed that SAA induced rapid upregulation of IL-33 secretion in human airway epithelial cells. To exclude the possibility that LPS contamination in the SAA preparation accounted for the induction of IL-33, SAA was incubated with polymyxine B (PMB), a cationic cyclic lipopeptide that forms a complex with LPS and blocks its biological effects. We found that PMB treatment did not significantly affect *IL33* mRNA induced by SAA (Figure 2E). Similarly, SAA-induced IL-33 secretion was not affected by PMB treatment (Figure 2F). Therefore, the observed increase of IL-33 production resulted primarily from SAA stimulation. Nuclear IL-33 functions as an alarm signal (alarmin) which is generally mediated by necrotic cell death [21]. To address whether IL-33 was released from necrotic cells, we analyzed membrane integrity by LDH activity. When the cells were exposed to medium or to SAA, LDH activity was undetectable in the supernatants. On the contrary, when the cells were exposed to Nonidet P-40 (NP-40), a significant release of LDH was observed (Figure 2G). Cell viability was also determined by Hoechst/PI staining assay. Hoechst dyes (blue) are cell-permeable and can bind to DNA in living cells, whereas PI dyes (red) are only permeable to dead cells. As shown in Figure 2H, only NP-40 treated cells showed significant cell death. Together, these results suggest that SAA induced IL-33 mRNA up-regulation and secretion levels without engaging cell necrosis. 

### 2.3. The MAP Kinases (p38/ERK1/2) and NF-κB Play a Critical Role in SAA-Induced IL-33 Secretion in Human Airway Epithelial Cells

**Figure 3:**
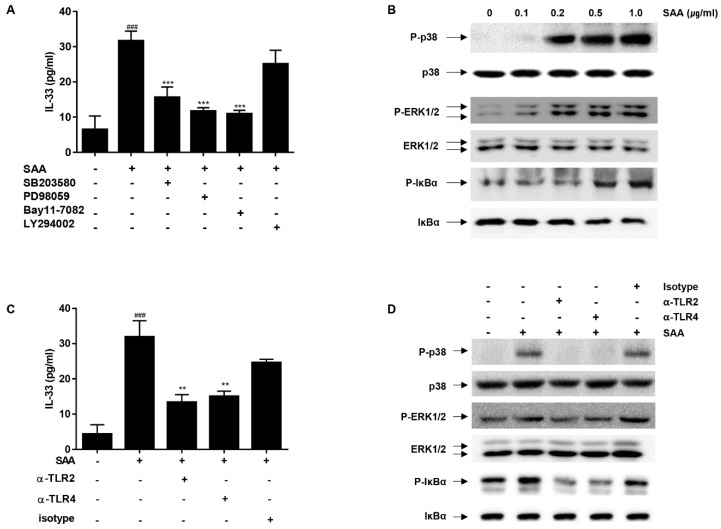
Signaling pathway and receptors involved in IL-33 secretion. (**A**) NCI-H292 cells were pretreated with SB203580 (10 μM), PD98059 (10 μM), Bay11-7082 (5 μM), and LY294002 (10 μM) individually for 30 min as indicated. Cells were then stimulated with 0.2 μg/mL of SAA for 6 h. After incubation, the levels of IL-33 protein in the supernatant were determined by ELISA. (**B**) NCI-H292 cells were stimulated with indicated SAA concentration for 15min. The phosphorylation of p38, ERK1/2, and IκBα was determined by western blotting, using various phospho-specific antibodies as indicated. Equal loading of samples was determined using antibodies against the unphosphorylated form of each protein. (**C**) NCI-H292 cells were incubated with neutralizing antibodies against TLR2 (α-TLR2, 1 μg/mL) or TLR4 (α-TLR4, 2 μg/mL) for 30min before stimulation with SAA (0.2 μg/mL). Isotype-matching IgG controls (1 μg/mL) were included. After 6 h, the levels of IL-33 protein in the supernatants were determined by ELISA. (**D**) α-TLR2 (1 μg/mL), α-TLR4 (2 μg/mL), or IgG-κ (1 μg/mL) were applied 30min before stimulation with SAA (0.5 μg/mL). After 15min, the phosphorylation of p38, ERK1/2, and IκBα were determined by Western blotting using phospho-specific antibodies of each molecule. Equal loading of samples was determined by antibodies against the unphosphorylated form of each molecule. ^###^ *p* < 0.001, compared with medium alone; **p < 0.01 and *** *p* < 0.001, compared with SAA alone.

MAP kinases, NF-κB activation and PI3K (phosphatidylinositol 3-kinase-Akt) are critical signaling components for the expression of inflammatory cytokines [22,23]. Therefore, we evaluated whether the activation of MAPKs, NF-κB and PI3K are involved in IL-33 secretion induced by SAA in NCI-H292. We employed specific inhibitors of MAP kinase signaling cascades; SB203580 (a p38 MAPK inhibitor), PD98059 (a MAPK ERK1/2, MEK-1 inhibitor), Bay11-7082 (an IKKβ inhibitor), and LY294002 (a PI3K inhibitor). SAA-induced IL-33 secretion was significantly blocked by SB203580, PD98059, and Bay 11-7082, whereas LY294002 showed no significant effect (Figure 3A). Next, we assessed the effects of SAA on the phosphorylation of these MAP kinases using Western blot analysis. As shown in Figure 3B, SAA strongly induced the phosphorylation of p38. Similarly, a gradual increase in the phosphorylation of ERK1/2 was seen at SAA concentrations up to 1.0 μg/mL. Since the activation of NF-κB represents a downstream event of p38/ERK1/2 signaling, we examined the phosphorylation of IκBα. As expected, SAA induced the phosphorylation of IκBα in a dose-dependent manner. These results suggest that p38, ERK1/2, and NF-κB play a role in SAA-induced IL-33 secretion.

### 2.4. IL-33 Secretion by SAA Is Mediated by TLR2/4 in Human Airway Epithelial Cells

Previously, several receptors, including TLR2 [24], TLR4 [25], and P2X_7_ [26], have been shown to contribute to SAA-mediated signaling. To identify the functional receptors responsible for SAA-induced IL-33 secretion in NCI-H292 cells, we applied neutralizing antibodies against TLR2 and TLR4 to the cells prior to SAA stimulation. As shown in Figure 3C, neutralizing antibodies against TLR2 and TLR4 effectively blocked SAA-induced IL-33 secretion. We also used oxidized ATP (oATP), a P2X_7_ signaling inhibitor. oATP did not inhibit SAA-mediated IL-33 secretion (Supplementary Figure S1). To further verify the involvement of TLR2/TLR4 receptors in SAA-dependent signaling, we performed Western blot analysis (Figure 3D). Treatment with either α-TLR2 or α-TLR4 resulted in a reduction in SAA-induced ERK1/2 and IκBα phosphorylation. Interestingly, α-TLR2 or α-TLR4 completely blocked p38 phosphorylation. Taken together, these results suggest that TLR2/4 are required for IL-33 secretion in response to SAA. 

### 2.5. Picroside II Inhibits SAA-Induced Expression/Secretion of IL-33 *via* TLR2 in Human Airway Epithelial Cells

**Figure 4:**
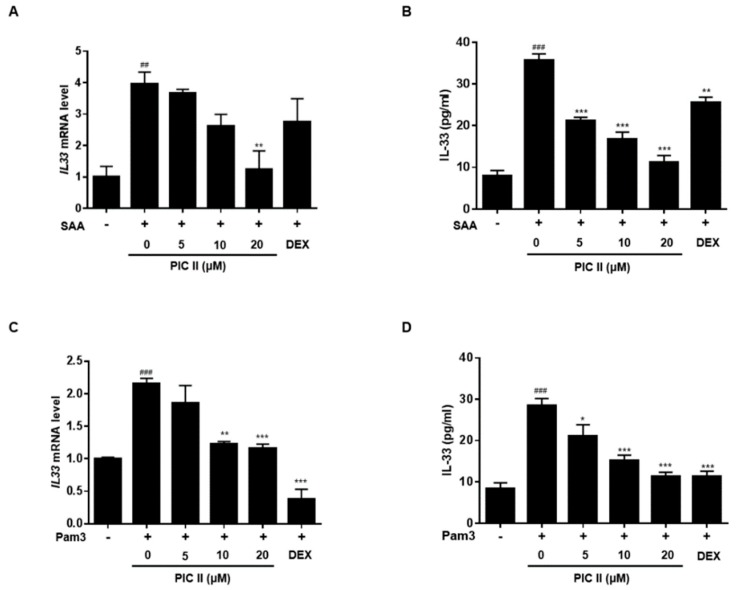
Picroside II inhibits SAA-induced IL-33 production via TLR2. (**A**) Effect of PIC II on the SAA-induced *IL33* mRNA expression was measured by qRT-PCR. NCI-H292 cells were pretreated with indicated concentrations of PIC II or DEX (1μM) for 1h and subsequently treated with SAA (0.5 μg/mL) for 6 h. (**B**) Effect of PIC II on the SAA-induced IL-33 secretion was assayed using ELISA. NCI-H292 cells were pretreated with PIC II or DEX (1 μM) for 1h and subsequently treated with SAA (0.2 μg/mL) for 6 h. (**C**) Effect of PIC II on the Pam3CSK4 (pam3)-induced *IL33* mRNA expression was measured by qRT-PCR. NCI-H292 cells were pretreated with corresponding concentrations of PIC II or DEX (1 μM) for 1h and subsequently treated with pam3 (0.2 μg/mL) for 6 h. (**D**) Effect of PIC II on the pam3-induced IL-33 secretion was assayed by ELISA. NCI-H292 cells were pretreated with PIC II or DEX (1 μM) for 1h and subsequently treated with pam3 (0.2 μg/mL) for 6 h. ^##^ *p* < 0.01 and ^###^ *p* < 0.001, compared with medium alone; * *p* < 0.01, ** *p* < 0.01 and *** *p* < 0.001, compared with SAA or Pam3 alone.

We next examined the suppressive effect of picroside II on the production of IL-33 in NCI-H292 cells. Cells were pretreated with picroside II for 1h and stimulated with SAA, and then the expression and secretion levels of IL-33 were measured by RT-qPCR and ELISA. As shown in Figure 4A, SAA-induced *IL33* mRNA was gradually decreased with the increasing dose of picroside II. Similar trends were observed in IL-33 protein secretion (Figure 4B). Picroside II and SAA used in this experiment did not exhibit any cytotoxic effect (Supplementary Figure S2). In contrast with picroside II, DEX did not affect SAA-induced *IL33* mRNA expression. Figure 3C showed that TLR2 is involved in SAA-mediated IL-33 secretion. To investigate the role of TLR2 in picroside II-mediated IL-33 suppression, we used synthetic TLR2 ligand Pam3CSK4 (Pam3). We observed that both picroside II and DEX effectively inhibited pam3-mediated IL-33 expression as well as secretion (Figure 4C,D). Similar IL-33 expression patterns were observed when we used β-actin as reference gene (Supplementary Figure S3A,B).

### 2.6. Picroside II Inhibits SAA-Induced Activation of MAP Kinase and NF-κB Pathway in Human Airway Epithelial Cells

**Figure 5:**
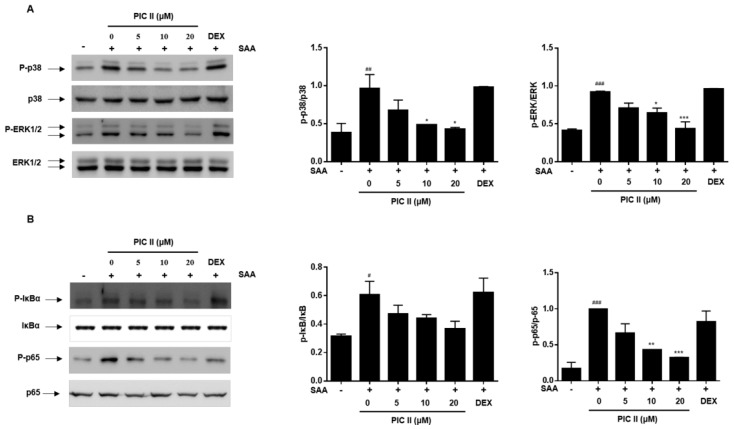
Picroside II inhibits SAA-induced activation of MAP kinases and NF-κB in NCI-H292 cells. NCI-H292 cells were pretreated with indicated concentrations of PIC II or DEX (1 μM) for 1h and subsequently treated with SAA (0.5 μg/mL) for 15 min. (**A**) MAPKs (p38 and ERK1/2) and (**B**) NF-κB (IκBα and p65) phosphorylation were determine by western blot analyses. The expression of unphosphorylated forms of MAPKs and NF-κB is shown as the lading control. The relative protein levels of phospho-p38/p38, phospho-ERK1/2/ERK1/2, phospho-IκBα/IκBα, and phosphor-p65/p65 were quantified using Image J software. ^#^ *p* < 0.05, ^##^ *p* < 0.01 and ^###^ *p* < 0.001, compared with medium alone; * *p* < 0.01, ** *p* < 0.01 and *** *p* < 0.001, compared with SAA alone.

In Figure 3, we showed that SAA-induced IL-33 production was mediated through MAPKs and NF-κB signals. Thus, we next examined whether inhibitory effects of picroside II exerted through p38, ERK1/2 and NF-κB signaling components using western blot analysis. The phosphorylation levels of p38, ERK1/2, IκBα and p65 were increased in SAA-stimulated NCI-H292 cells (Figure 5). Treatment with picroside II resulted in a reduction of SAA-induced p38-ERK1/2, IκBα and p65 phosphorylation in a dose-dependent manner. However, DEX did not affect the activation of these signaling molecules.

Taken together, our data indicated that picroside II inhibits SAA expression induced by LPS in human monocytes. Moreover, picroside II effectively inhibits SAA-induced IL-33 productions through TLR2-derived p38/ERK1/2 and NF-κB signaling pathways in airway epithelial cells.

## 3. Discussion

It has been shown that SAA/IL-33 play a role in the pathology of steroid-resistant lung inflammation [6,15]. In present study, we examined whether picroside II affects *SAA1* expression in monocytes and SAA-induced IL-33 production in airway epithelial cells. We showed that picroside II affected SAA-dependent pathways at multiple levels. Picroside II down-regulated not only LPS- but also LPS plus DEX-induced *SAA1* mRNA in human monocytes. Although we clearly demonstrated that picroside II inhibited the LPS-induced SAA expression, further studies are needed to verify that it also inhibits SAA protein secretion. It would be interesting to investigate the effects of picroside II on macrophage cells derived from COPD patients.

It has been reported that SAA induces *IL33* gene expression in human monocytes and mouse macrophages [27]. However, whether SAA can induce IL-33 secretion from airway epithelial cells remains to be investigated. Our results showed that SAA induced IL-33 secretion effectively and rapidly, whereas SAA minimally affected *IL33* mRNA expression. IL-33 is constitutive expressed at high levels and stored in the nucleus of endothelial and epithelial cells [28]. These results indicate that SAA may lead the secretion of preformed IL-33 protein than gene induction in human airway epithelial cells. In line with this, it was reported that allergen (fungal allergen *Alternaria alternate* extract)-induced IL-33 release was not associated with significant induction of *IL33* mRNA in normal human bronchial epithelial (NHBE) cells [29]. Thus it is interesting to speculate that SAA could stimulate rapid secretion of existing IL-33 to cope with immediate needs as an ‘alarmin’ in epithelial cells. To date, only few reports show the IL-33 release by epithelial cells after certain stimulation. Exposure to ragweed pollen [30] or airway uric acid [31] induced the extracellular release of IL-33 from epithelial cells. IL-33 is also released during necrotic cell death [21]. We could not detect LDH activity in the cells exposed to SAA. Therefore, it is unlikely that cell necrosis is associated with SAA-induced secretion of IL-33 in lung epithelial cells. 

SAA has cytokine-inducing properties via multiple receptors including TLR2, TLR4, formyl peptide receptor-like 1 (FPRL1), and P2X_7_ receptor (P2X_7_R) [32]. Our results using neutralizing antibodies against TLR2/TLR4 suggest that SAA is an endogenous ligand for TLR2/4 in SAA-induced secretion of IL-33. TLRs are key players of the innate immune system, playing an important role in the detection of danger signaling molecules. Functional TLR2 and TLR4 have been detected on NCI-H292cells [33]. Indeed, the synthetic TLR2 ligand Pam 3CSK4 (Pam3)-induce IL-33 production was completely suppressed by picroside II. Currently, we cannot discriminate the exact target of picroside II in the SAA signaling pathways. Further studies using agonists and antagonists of each signaling pathway involved in SAA-induced responses will have to be conducted to determine picroside II’s target.

Our results indicated that picroside II exerted inhibitory effects by attenuating p38-ERK1/2 MAPK and NF-κB activation. NF-κB regulates expression of various inflammatory cytokines including TNF-α [34]. Thus, one might expect that picroside II would also inhibit TNFα expression. Interestingly, however, picroside II did not inhibit TNFα expression, while DEX did effectively. Further study will be required to elucidate the precise mechanism of SAA and IL-33 inhibition by picroside II, nevertheless our results clearly demonstrated that picroside II is a potent inhibitor of SAA and IL-33 production, which is resistant to DEX. Given the fact that SAA and IL-33 involved in many biological processes [7,35], our findings will provoke further studies exploring the effects of picroside II in other diseases associated with SAA and IL-33.

## 4. Materials and Methods

### 4.1. Reagents and Antibodies

Recombinant human SAA was purchased from Peprotech EC (Rocky Hill, NJ, USA). The functional grade anti-TLR2, anti-TLR4 and their isotype control IgG were purchased from Biolegend (San Diego, CA, USA) and the pam3CSK4 was bought from Invivogen (San Diego, CA, USA). Lipopolysaccharide (LPS; from *Escherichia coli* strain 055:B5), dexamethasone (DEX), Bay 11-7082, PD98059, LY294002, SB203580, oATP, polymyxin B (PMB), nonidet P-40 and all other chemicals were obtained from Sigma-Aldrich (St. Louis, MO, USA). Antibody against phospho-ERK1/2, phospho-p38, and p38 were obtained from Cell Signaling Technology (Danvers, MA, USA). Antibody against ERK1/2, phospho-IκBα, IκBα, phosphor-p65, p65 and secondary antibodies were obtained from Santa Cruz (Dallas, TX, USA).

### 4.2. Preparation of Picroside II

Picroside II (PIC II, Figure 1A) was purified as described previously and purity was more than 99.5% as determined by ultra-performance liquid chromatography [19]. The Picroside II peak areas from the UPLC chromatogram at 254 nm were integrated using the processing software Empower 2 (Waters Corporation, Milford, MA, USA). The integrated peak areas were substituted on a standard calibration curve linear model to determine protein yield and to conduct comparative analysis. The standard calibration curves were obtained by dissolving standard compounds in methanol to generate eight or nine concentration data points ranging from 0 to 100 μg/mL. Each calibration standard was injected in triplicate into an ACQUITY UPLC (Waters Corporation) system equipped with a photodiode array (PDA, Waters Corporation) to confirm the precision and repeatability of the validated method within a single day. The linearity of the developed method was evaluated at eight or nine concentration levels from 0.1 to 100 μg/mL.

### 4.3. Cell Culture 

Human airway epithelial cell line NCI-H292 and human monocytic leukemia cell line THP-1 were purchased from the American Type Culture Collection (ATCC, Manassas, VA, USA). Cells were grown in RPMI 1640 supplemented with 10% fetal bovine serum (Gibco Inc., Brooklyn, NY, USA) in the presence of penicillin plus streptomycin (Gibco Inc.), and 25mM of HEPES at 37 °C in a humidified with 5% CO_2_. Cells were split every three or four days and were used within 20 passages. All cell cultures were tested and determined to be free of mycoplasma contamination (e-Myco™ Mycoplasma PCR Detection kit, iNtRON Biotechnology, Seongnam, Korea). Cells at a confluence of 60-80% were stimulated with the experimental reagents in serum-free medium as indicated in each experiment. 

### 4.4. Cell Survival Assay

To test the cytotoxicity, NCI-H292 cells were treated with SAA or 0.5% Nonidet P-40 for 6 h. Then, lactate dehydrogenase (LDH) activity in the supernatants was measured using a cytotoxicity detection kit (Roche, Indianapolis, IN) following the manufacturer’s instructions. Cell viability was also assessed by the use of Hoechst 33,342 (Hoechst, Invitrogen, Carlsbad, CA, USA) and propidium iodide (PI, Invitrogen) double staining in NCI-H292 cells. Cells treated with SAA for 6 h or 0.01% NP-40 for 30 min were collected and stained with Hoechst 33,342 (25 μg/mL) and PI (5 μg /mL) for 5 min at 37 °C. The images were obtained using a fluorescence photomicroscope (Nikon Corp., Tokyo, Japan).

### 4.5. Enzyme-Linked Immunosorbent Assay (ELISA)

The level of IL-33 release in the culture supernatants was determined by commercially available ELISA kit (R&D Systems, Minneapolis, MN, USA) according to the manufacturer’s instructions. 

### 4.6. RNA Extraction, RT-PCR and Quantitative Real-Time RT-PCR

Total RNA extraction and reverse transcription was performed as described previously [19]. Quantitative real-time RT-PCR was performed using iQ SYBR Green supermix (Bio-Rad, Hercules, CA). The mRNA levels of each target gene was normalized to the levels of *GAPDH* mRNA. The primer sequences were as follows: *GAPDH* (forward, 5′-CCTGCACCACCAACTGCTTA-3′, reverse, 5′-GTCTTCTGGGTGGCAGTGAT); *β-actin* (forward, 5′-TCCAGCCTTCCTTCCTGGGCAT, reverse, 5′-CTTGATCTTCATTGTGCTGGGTGCC); *IL33* (forward, 5′-GGTGTTACTGAGTTACTATGAG-3′, reverse, 5′-GGAGCTCCACAGAGT GTT CCTTG-3′); *SAA1* (forward, 5′-GTGATCAGCGATGCCA GAGA-3′, reverse, 5′-TACCCTCTCCCCGCTTTGTA-3′); and *TNF* (forward, 5′-CCGAGTGACAAG CCTGTAGC-3′, reverse, 5′- GAGGACCTGGGAGTAGATGAG-3′). 

### 4.7. Western Blotting

Cells were lysed in lysis buffer containing protease inhibitor cocktail (Thermo Fisher Scientific, Rockford, IL, USA) and a phosphatase inhibitor mixture (Roche, Indianapolis, IN, USA). Total cellular protein were fractionated on 10% SDS-polyacrylamide gels and transferred to a polyvinylidene fluoride membranes. The membrane was incubated for 1h in 5% skim milk in TBS-T buffer (0.1M Tris-HCl, pH 7.4, 0.9% NaCl, 0.05% Tween-20), followed by an overnight incubation at 4 °C with primary antibodies. After washing, the membranes were incubated with appropriate secondary antibodies. The blot was developed using an ECL kit (Thermo Fisher Scientific). 

### 4.8. Statistical Analysis

All results were expressed as means ±SEMs of three independent experiments. Data were analyzed and graphed with GraphPad Prism software (version 6.07). Statistical analysis was calculated using analysis of variance (ANOVA) followed by a multiple comparison test with Dunnet’s adjustment. Statistical significance was accepted at a *P* values of less than 0.05. 

## 5. Conclusions

In conclusion, we have successfully demonstrated that picroside II inhibited LPS-induced *SAA1* mRNA expression in human monocytes and SAA-induced IL-33 secretion in human airway epithelial cells. We further demonstrated that picroside II attenuated SAA-induced IL-33 production by blocking TLR2-p38-ERK1/2-NF-κB signaling pathway. The results may provide a valuable information for the development of picroside II as a potential alternative therapeutic agent for steroid-resistant COPD.

## Supplementary Materials

The following are available online, Figures S1–S3.

## Abbreviations

COPD	chronic obstructive pulmonary disease
DEX	dexamethason
LDH	lactate dehydrogenase
LPS	lipopolysaccharide
MAPK	mitogen-activated protein kinase
NF-κB	nuclear factor-κB
Pam3	Pam 3CSK4
PI3K	phosphatidylinositol 3-kinase-Akt
PMB	polymyxin B
SAA	serum amyloid A
