# Supplementary material for: Picroside II Isolated from Pseudolysimachion rotundum var. subintegrum Inhibits Glucocorticoid Refractory Serum Amyloid A (SAA) Expression and SAA-induced IL-33 Secretion
Source: Molecules. 2019 May 27;24(10):2020. doi: 10.3390/molecules24102020 (PMC6572537; doi:10.3390/molecules24102020)
Supplement: Supplementary file 1 [file molecules-24-02020-s001.pdf]

Picroside II inhibits glucocorticoid refractory Serum Amyloid A (SAA) expression and SAA-induced IL-33 secretion

*Corresponding author (Hyun-Jun Lee)

Tel: +82-43-240-6115

Fax: +82-43-240-3464

Email: hjlee7@kribb.re.kr

Supplementary figure files

Supplementary Fig. S1. The inhibitory effect of picroside II on LPS-induced SAA expression in THP-1 cells.

Supplementary Fig. S2. Effects of picroside II on SAA-stimulated NCI-H292 cells viability.

Supplementary Fig. S3. Effect of PIC II on the SAA-induced IL33 mRNA expression in NCI-H292 cells.

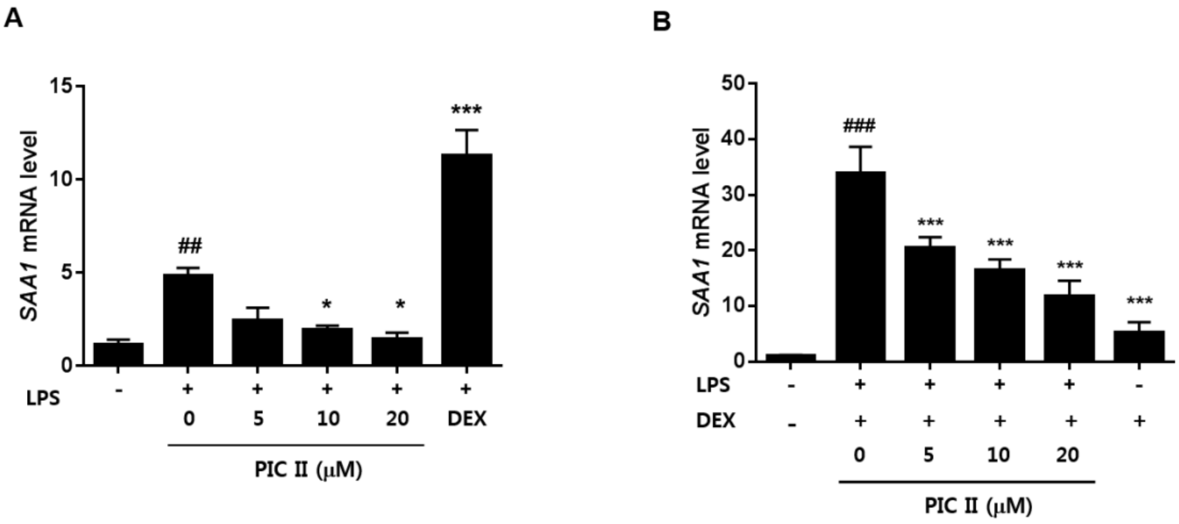

Supplementary Fig. S1. The inhibitory effect of picroside II on LPS-induced SAA expression in THP-1 cells.

THP-1 cells were pretreated with indicated concentrations of PIC II or DEX (1 μM) for 1h and then stimulated with LPS (0.2 ㎍/㎖) or LPS (0.2 ㎍/㎖) plus DEX (1 μM) for 6h. The mRNA expression of SAA1 was measured by real-time RT-PCR levels of *SAA1* gene expression were normalized to β-actin. ##p<0.01 and ###p<0.001, compared with medium alone;*p<0.05, and ***p<0.001, compared with LPS alone.

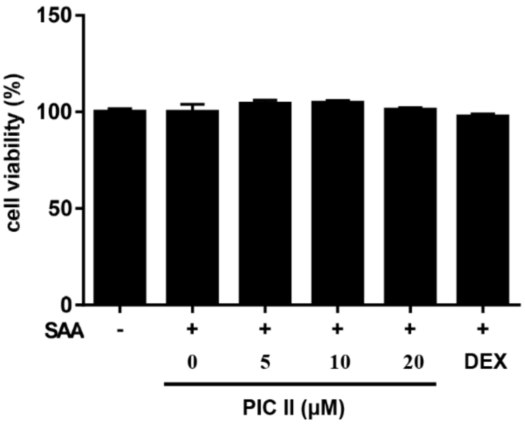

Supplementary Fig. S2. Effects of picroside II on SAA-stimulated NCI-H292 cells viability. Cells were pretreated with PIC II (5, 10, 20 μM) or DEX (1 μM) for 1h and stimulated with SAA (0.2 ㎍/㎖) for 24h. Cell viability was measured by means of a 3-(4,5-dimethylthiazol-2-yl)-2,5-diphenyltetrazolium bromide (MTT; Amresco, Solon, OH) assay.

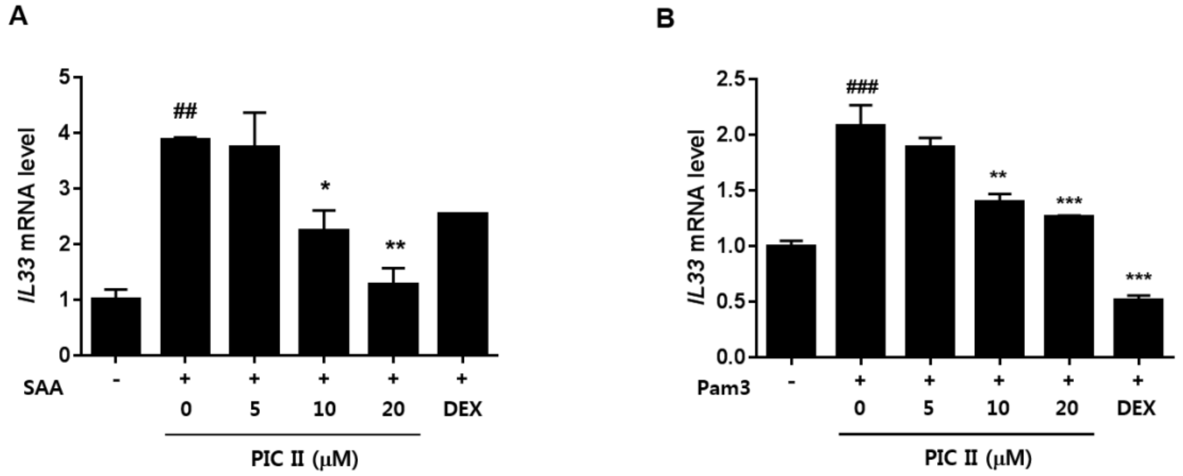

Supplementary Fig. S3. Effect of PIC II on the SAA-induced *IL33* mRNA expression in NCI-H292 cells.

NCI-H292 cells were pretreated with indicated concentrations of PIC II or DEX (1μM) for 1h and subsequently treated with (A) SAA (0.5 ㎍/㎖) or (B) pam3 (0.2 ㎍/㎖) for 6h. The mRNA expression of *IL33* was measured by real-time RT-PCR and levels of *IL33* gene expression were normalized to β-actin. ##p<0.01 and ###p<0.001, compared with medium alone; *p<0.01, **p<0.01 and ***p<0.001, compared with SAA or Pam3 alone.
